# Postoperative Intraocular Lens Position and Refractive Stability in Cataract Surgery: Influence of Target Refraction and IOL Design

**DOI:** 10.3390/medicina62071386

**Published:** 2026-07-17

**Authors:** Freja Bagatin, Renata Iveković, Ivana Barbić Radman, Karla Ranđelović, Zoran Vatavuk

**Affiliations:** 1 Department of Ophthalmology, Sestre Milosrdnice University Hospital Centre, 10000 Zagreb, Croatia; renata.ivekovic@kbcsm.hr (R.I.); ivana.radman@kbcsm.hr (I.B.R.); karla.randelovic@kbcsm.hr (K.R.); zoran.vatavuk@kbcsm.hr (Z.V.); 2 School of Dental Medicine, University of Zagreb, 10000 Zagreb, Croatia

**Keywords:** cataract surgery, monofocal intraocular lenses, target refraction, postoperative refractive shift, IOL stability

## Abstract

*Background and Objectives:* Achieving a postoperative refractive outcome close to the target is crucial in cataract surgery, as residual refractive errors can affect visual quality and patient satisfaction. *Methods:* This study evaluates the relationship between preoperative target refraction, postoperative intraocular lens (IOL) shift, and refractive stability. Prospective, observational study conducted at the Clinical Hospital Centre Sestre Milosrdnice, Zagreb, Croatia, involving patients undergoing routine cataract surgery with monofocal IOL implantation. Ninety-four eyes undergoing phacoemulsification with implantation of one of three monofocal IOLs (PCB00, RayOne HA, Akreos Adapt) were included. Preoperative biometry assessed axial length, anterior chamber depth, central corneal thickness, and white-to-white distance. Target refraction was −0.75 to 0.00 D. IOL position and refraction were measured at 1 week, 1 month, and 6 months postoperatively. Patient satisfaction was evaluated at all three time points. *Results:* All three IOLs demonstrated comparable in-the-bag stability over 6 months, with minimal longitudinal shift (all *p* > 0.05). Postoperative refractive outcomes were generally comparable, but RayOne HA showed a slightly more myopic deviation, whereas PCB00 and Akreos Adapt remained closer to emmetropia. The differences reflect the extent of deviation from the intended postoperative refraction, with statistically significant intergroup differences at 1 month (*p* = 0.004) and 6 months (*p* = 0.045). *Conclusions:* All three IOLs provided stable positioning and excellent visual outcomes. However, small differences in refractive predictability suggest that IOL design may influence the accuracy of target refraction. Tailoring target refraction according to IOL type may improve the likelihood of achieving emmetropia and optimize patient satisfaction.

## 1. Introduction

Achieving a postoperative refractive outcome close to the intended target is a central goal of modern cataract surgery, as residual refractive errors and astigmatism can significantly affect visual quality and patient satisfaction [1]. Even with precise biometry and careful IOL power calculation, small deviations from the target refraction remain common and are influenced by multiple factors, including anatomical changes after surgery, intraocular lens (IOL) design, and the accuracy of effective lens position (ELP) prediction [2]. The European Registry of Quality Outcomes reported that 73.7% of eyes had a refractive prediction error within ±0.5 D after cataract surgery [3]. It has been reported that a forward movement of the IOL by approximately 0.29 mm along the visual axis has been associated with a myopic shift of −0.4 D [4]. Wang and colleagues reported that 0.5 mm decentration of an aspheric IOL could eliminate its aberration-correcting effect [5].

Postoperative refractive changes are often associated with dynamic shifts in IOL position, particularly during the early postoperative period, as the lens settles within the capsular bag [6,7]. Accurate measurement of ELP is therefore essential for understanding the factors contributing to refractive outcomes. Scheimpflug imaging provides precise, non-contact measurements of the axial IOL position along the visual axis and has been successfully used in prior studies, including Na et al., to assess longitudinal lens stability [8].

Different monofocal IOL designs may exhibit subtle variations in postoperative behavior due to differences in material, haptic configuration, and interaction with the capsular bag, which can influence both refractive stability and predictability [9]. The choice of target refraction, such as a slight myopic bias, may be tailored according to the specific IOL type to optimize visual outcomes and minimize the risk of residual hyperopia [10].

This study aimed to evaluate the positional stability and refractive outcomes of three commonly used monofocal IOLs—PCB00, RayOne HA, and Akreos Adapt—over the first six postoperative months. Using Scheimpflug imaging, we assessed axial IOL shifts and correlated these with deviations from the intended target refraction, as well as patient-reported satisfaction, to better understand the factors influencing postoperative refractive predictability.

## 2. Materials and Methods

This prospective observational study was conducted from May 2024 to October 2025 at the Department of Ophthalmology, Clinical Hospital Center Sestre Milosrdnice, Zagreb. The study included 94 patients undergoing routine cataract surgery with implantation of a monofocal IOL.

A total of 105 consecutive eligible patients undergoing routine cataract surgery with implantation of a monofocal intraocular lens (IOL) were prospectively enrolled during the predefined study period. Patients who did not complete the scheduled 6-month follow-up were excluded from the final analysis. Consequently, 94 patients completed the study and were included in the final analysis. As this was an exploratory prospective observational study, no formal a priori sample size calculation was performed. Patients with age-related cataract scheduled for standard phacoemulsification surgery were eligible for inclusion. Exclusion criteria included previous ocular surgery, ocular diseases (such as corneal pathologies, uveitis, zonal instabilities, pseudoexfoliation, glaucoma or retinal disease affecting visual acuity), irregular astigmatism, intraoperative complications or postoperative conditions that could influence refractive outcomes. Additionally, those patients who failed to return for scheduled follow-ups were also excluded. Eyes with extreme refractive errors were excluded to ensure uniform IOL targeting (IOL power < 16 D or >27 D). Only one eye per patient was included in the analysis.

All patients underwent a comprehensive preoperative ophthalmic examination. Nucleus grading was classified according to Lens Opacities Classification System III by the same specialist. Preoperative biometric measurements, including axial length (AL), anterior chamber depth (ACD), lens thickness (LT), white-to-white (WTW) distance and keratometry, were obtained using optical biometry with the IOLMaster 700 (Carl Zeiss Meditec, Jena, Germany). Additional parameters such as central corneal thickness (CCT), intraocular pressure (IOP), and best-corrected visual acuity (BCVA) were also recorded. These measurements were used to calculate the appropriate IOL power and to determine the target postoperative refraction. The SRK/T formula was used due to its proven accuracy in eyes with average axial lengths and its widespread use for predicting postoperative emmetropia [11]. The target postoperative refraction was set from slight myopia to emmetropia (−0.75 D to 0.00 D), with slight myopia generally preferred in routine clinical practice to reduce the risk of postoperative hyperopia.

All cataract surgeries were performed using standard phacoemulsification through a superior clear corneal 2.7 mm incision with implantation of a foldable monofocal IOL into the capsular bag. The surgical procedure included 5.5 mm continuous curvilinear capsulorhexis, hydrodissection, phacoemulsification of the nucleus, irrigation/aspiration of the remaining cortex, in-the-bag implantation of the IOL and final hydration of the incisions. The selection of the IOL model was performed according to standard clinical practice, taking into account lens availability, without specific patient-related selection criteria. All procedures were performed by the same experienced cataract surgeon to minimize variability. The sample was divided into three groups based on the implanted IOL: Group 1 (PCB00), Group 2 (Akreos Adapt), and Group 3 (RayOne HA). Key characteristics of each IOL relevant to in-bag stability and ELP are summarized in Table 1.

Postoperative topical therapy included 0.1% dexamethasone eye drops (Maxidex, Alcon Laboratories, Inc., Fort Worth, Texas, USA) administered four times daily, and dexamethasone eye ointment (Dexa-Gel, Ursapharm Arzneimittel GmbH, Saarbrücken, Germany) applied once nightly for one month.

Postoperative examinations were performed at 1 week, 1 month, and 6 months. At each visit, uncorrected (UCVA) and best-corrected distance visual acuity (BCVA), manifest refraction, spherical equivalent (SE), and slit-lamp biomicroscopy were assessed. Visual acuity was measured monocularly using Snellen charts at a distance of 6 m, and BCVA was determined after manifest (subjective) refraction as the best visual acuity achieved with optimal refractive correction. The spherical equivalent was calculated as the sum of the spherical component plus half of the cylindrical component of the manifest refraction, and was used to summarize the overall refractive error of the eye.

Anterior segment imaging with the Pentacam (Oculus Optikgeräte, Wetzlar, Germany) was performed at each postoperative visit to assess IOL position and anterior segment parameters. IOL position was manually measured on Scheimpflug images acquired with the Pentacam HR at the 180° meridian. Using the software’s manual caliper tool, the distance was measured from the corneal apex, automatically identified by the Pentacam1.29r12-1 software, to the center of the anterior hyperreflective IOL signal approximating the visual axis. To ensure consistency across follow-up visits, all measurements were performed using the device’s built-in reference lines and the same measurement protocol. Longitudinal IOL shift was calculated as the change in this distance between postoperative visits (1 week to 1 month, and 1 month to 6 months), with positive values indicating forward displacement and negative values indicating backward movement. The margin of error of postoperative refraction was defined as the difference between the measured SE and the target refraction for each IOL type. Positive values indicate a hyperopic shift relative to the target, whereas negative values indicate a myopic shift. These refractive deviations were analyzed in relation to IOL positional changes to evaluate the effect of longitudinal IOL shift on postoperative refractive outcomes.

Primary outcomes included postoperative SE, deviation from the target refraction (margin of error), and IOL positional changes (IOL shift) during follow-up. Secondary outcomes included the proportion of eyes achieving refractive outcomes within ±0.50 D of the target and patient satisfaction with visual outcomes.

Normality of data distribution was analyzed with Kolmogorov–Smirnov test and appropriate parametric or non-parametric tests were used accordingly. Baseline characteristics were analyzed using one-way ANOVA followed, where appropriate, by Scheffé’s post hoc test, Pearson’s chi-squared test for categorical values, while differences between two groups were assessed by t-test for independent samples. Bar charts with 95% confidence intervals were used in graphical representation of significant differences. All P values below 0.05 were considered significant. MedCalc^®^ Statistical Software version 20.218 (MedCalc Software Ltd., Ostend, Belgium; https://www.medcalc.org; 2023, (accessed on 14 July 2026).) was used in all statistical procedures.

This study adhered to the tenets of the Declaration of Helsinki, and ethical approval was obtained from the Institutional Review Board of the Sestre milosrdnice University Hospital Center (approval number: 003-06/24-03/026, 9 April 2024). Written informed consent was obtained from all participants before enrolment in the study.

## 3. Results

The study included 94 patients undergoing cataract surgery with ages ranging from 54 to 93 (mean age 75.3 years). The sample was divided into three groups according to the implanted IOL: PCB00, Akreos Adapt and the RayOne HA IOL group.

Baseline characteristics and differences between groups are shown in Table 2. There was no statistically significant difference between the three groups regarding age, gender and preoperative biometric parameters, as well as target refraction.

The positional stability of the three IOLs—PCB00, Akreos Adapt, and RayOne HA—was evaluated at 1 week, 1 month, and 6 months postoperatively, along with the longitudinal positional shifts between these time points (Table 3). At 1 week, mean IOL positions ranged from 3990 ± 259.8 µm (Akreos Adapt) to 4116.7 ± 276.4 µm (RayOne HA) and 4136.7 ± 391.1 µm (PCB00), with no statistically significant differences among the groups (*p* = 0.149). By 1 month, mean positions remained similar across IOL types (PCB00: 4005.2 ± 752.5 µm; Akreos Adapt: 4015.2 ± 313.0 µm; RayOne HA: 4104.8 ± 311.8 µm), again without significant intergroup differences (*p* = 0.730). At 6 months, all lenses maintained stable in-the-bag positions (PCB00: 4045.0 ± 286.6 µm; Akreos Adapt: 3953.5 ± 305.4 µm; RayOne HA: 4069.3 ± 315.9 µm), and no significant differences were observed (*p* = 0.295).

**Figure 1:**
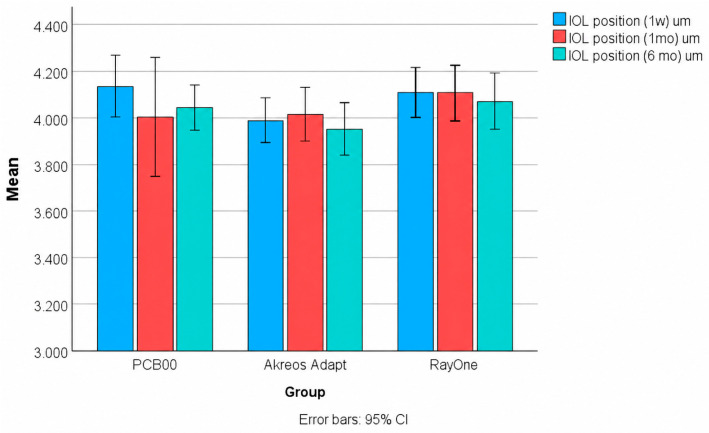
In-the-bag stability of 3 types of IOLs at 1 week, 1 month, and 6 months.

Analysis of longitudinal IOL shift revealed minor changes over time. Between 1 week and 1 month, PCB00 exhibited a small anterior shift (+35.3 ± 212.4 µm), RayOne HA showed a minimal anterior shift (+17.0 ± 184.6 µm), and Akreos Adapt moved slightly posteriorly (−24.8 ± 259.0 µm), with these differences not reaching statistical significance (*p* = 0.535). From 1 month to 6 months, all IOL types demonstrated small anterior shifts (PCB00: +80.0 ± 241.8 µm; RayOne HA: +35.6 ± 243.3 µm; Akreos Adapt: +73.2 ± 293.0 µm), again without significant intergroup differences (*p* = 0.781), indicating overall good positional stability of all lenses over the six-month follow-up period (Table 3, Figure 1).

There were no significant differences in IOL shifts after one week, one month and six months.

Postoperative refractive outcomes among the three IOL groups during the follow-up period are presented in Table 4. At 1 week postoperatively, the mean SE was −0.2 ± 0.6 D in the PCB00 group, −0.2 ± 0.4 D in the Akreos Adapt group, and −0.4 ± 0.8 D in the RayOne HA group, with no statistically significant difference between the groups (*p* = 0.296). Similar refractive outcomes were observed at 1 month and 6 months postoperatively. At 6 months, the mean SE was −0.2 ± 0.5 D, −0.1 ± 0.6 D, and −0.3 ± 0.7 D in the PCB00, Akreos Adapt, and RayOne HA groups, respectively (*p* = 0.156).

Mean postoperative UCVA improved in all groups during follow-up. At 1 week postoperatively, the mean UCVA was 0.7 in the PCB00 group, 0.8 in the Akreos Adapt group, and 0.7 in the RayOne HA group. At 6 months, the corresponding mean UCVA values were 0.8, 0.8, and 0.7, respectively.

Table 5 shows how far we deviated from target refraction. The margin of error of postoperative refraction (PR), which was calculated as the postoperative refraction (SE) minus the target refraction in three different types of IOLs, was analyzed at 1 week, 1 month and 6 months after surgery.

A positive margin of error value indicates that the PR showed a hyperopic shift compared to the target refraction, whereas a negative margin of error value indicated that the PR showed a myopic shift compared to the target refraction.

**Figure 2:**
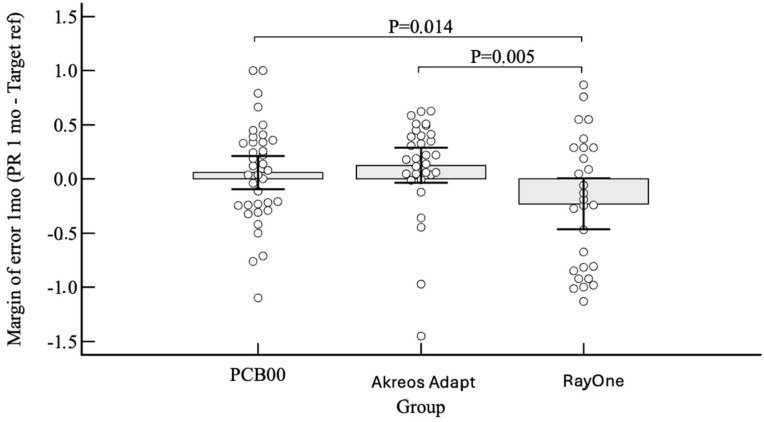
Differences in the margin of error of PR after 1 month: Scheffé’s post hoc test; marked are only groups with significant differences.

Postoperative refractive outcomes showed slightly positive deviations for PCB00 and Akreos Adapt (≈+0.1 to +0.2 D), indicating patients ended closer to emmetropia or mildly hyperopic, whereas RayOne HA tended toward slightly myopic outcomes (≈−0.3 to −0.4 D). Statistically significant differences among the IOL groups were observed at 1 month (*p* = 0.004) and 6 months (*p* = 0.045 (Table 5, Figure 2).

**Figure 3:**
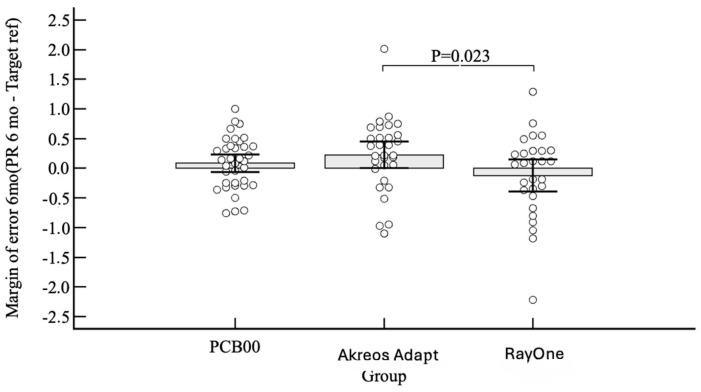
Differences in the margin of error of PR after 6 months: Scheffé’s post hoc test; marked are only groups with significant differences.

There were significant differences in margin of error after 6 months (*p* = 0.045). The best values (closest to 0) were among the PCB00 group, while Akreos Adapt had significantly highest values in positive shift compared to Rayner group (*p* = 0.023) (Figure 3). Additionally, the proportion of eyes within ±0.50 D and ±1.00 D of the target refraction at final follow-up is presented in Appendix A, Table A1.

Patient satisfaction was high across all IOL groups, with no statistically significant differences at 1 month (*p* = 0.059) or 6 months (*p* = 0.978) and a trend toward slightly lower satisfaction in the RayOne HA group at 1 month that equalized over time (Table 6).

## 4. Discussion

The present study evaluated the positional stability and refractive outcomes of three different monofocal IOLs following uncomplicated phacoemulsification. The main findings indicate that all three IOL types demonstrated comparable in-the-bag stability over time, with minimal longitudinal shift between postoperative visits. However, small but consistent differences in postoperative refractive outcomes were observed, suggesting that IOL design and ELP prediction may influence refractive predictability independent of positional stability and that target refraction may need to be adjusted according to the implanted lens type.

Early postoperative refractive changes, characterized by an initial hyperopic tendency followed by a myopic shift within the first postoperative week, have been reported in the literature. This phenomenon is most likely related to dynamic changes in ACD and ELP, as the IOL may initially be positioned slightly more posteriorly immediately after implantation and subsequently move anteriorly during the early postoperative period. These positional changes, together with postoperative corneal alterations, can contribute to transient refractive instability before stabilization occurs [6]. In addition to dynamic postoperative changes in ACD and ELP, transient alterations in corneal power related to corneal incision healing and postoperative corneal edema may also contribute to the early refractive fluctuations observed after cataract surgery. These corneal changes are generally most pronounced during the first postoperative days and tend to stabilize within the first few weeks, after which the refractive outcome is predominantly determined by IOL position and biometric factors [12]. Previous studies have further demonstrated that IOLs typically undergo anterior positional changes during the first postoperative week, followed by relative stabilization, which corresponds with early refractive fluctuations. Refractive outcomes have been reported to stabilize as early as 1 week, with no significant differences compared to 4 weeks, although most patients achieve full stability by 4 weeks postoperatively [7]. These findings are consistent with our results, showing small anterior–posterior IOL shifts over time, followed by relative refractive and positional stability at later follow-ups.

Even with precise biometry and careful IOL power calculation, residual refractive errors after cataract surgery remain common and are influenced by multiple factors. Sources of refractive error include inaccurate prediction of the ELP, subtle anatomical changes induced by surgery, and IOL design-specific characteristics. Uncomplicated cataract surgery has been shown to induce significant modifications in several ocular dimensions: ACD typically increases, LT decreases, and the iridocorneal angle may widen postoperatively. These anatomical changes can lead to errors in ELP prediction, which have been reported to account for approximately 22–38% of total refractive prediction error [7,13]. Furthermore, the axial position of the IOL may continue to shift in the first months after surgery due to capsular bag fibrosis and contraction, and even small displacements can significantly alter the postoperative SE [14].

From 1 month to 6 months, all IOL types demonstrated small anterior shifts (PCB00: +80.0 ± 241.8 µm; RayOne HA: +35.6 ± 243.3 µm; Akreos Adapt: +73.2 ± 293.0 µm), without significant intergroup differences (*p* = 0.781). These results indicate that all evaluated IOLs provide stable fixation within the capsular bag, with minor clinically relevant movement over time. In our cohort, the early myopic or hyperopic shifts corresponded closely with these measured anterior–posterior IOL movements, confirming that initial postoperative refractive instability is primarily driven by dynamic changes in lens position rather than long-term instability, with refractive outcomes stabilizing by 1 month and remaining consistent through 6 months. This is consistent with the existing literature demonstrating excellent postoperative stability of modern monofocal and toric IOL designs, with minimal positional change and limited impact on visual performance [7,8,15].

Although slight anterior and posterior movements were observed within each group, the overall magnitude of shift remained limited, typically within a range that is unlikely to induce significant refractive change. This supports the concept that postoperative capsular bag contraction and fibrosis lead to early stabilization of the IOL position, usually within the first postoperative weeks, as described in prior studies [8,16].

Fukumitsu et al. reported lens-specific differences in postoperative IOL movement, with Akreos MI-60 lenses demonstrating a posterior shift, while AcrySof IOLs tended to move anteriorly. However, despite these directional differences, the overall magnitude of movement was small and of limited clinical significance, which is consistent with our findings of minimal longitudinal IOL shift across all studied IOLs [17].

The findings of the present study are in agreement with recent work reporting minimal postoperative change in IOL orientation and position [15]. Moreover, studies examining ELP have highlighted that postoperative refractive error can arise from discrepancies between predicted and achieved lens position, independent of detectable IOL shift [10,16].

Despite the absence of significant differences in IOL position and shift, differences in postoperative refractive outcomes were observed. The RayOne HA IOL consistently showed slightly more myopic SE values at all postoperative time points compared with PCB00 and Akreos Adapt, which tended to be closer to plano or exhibited a mild hyperopic shift. Correspondingly, mean UCVA was slightly lower in the RayOne HA group (0.7 ± 0.3) than in the other groups (0.8 ± 0.2), although this difference was not statistically significant and did not affect BCVA, which reached 1.0 ± 0.1 in all groups.

An important aim of this study was to evaluate how effectively our habitual “first minus” targeting strategy achieves the desired postoperative refraction. While the ultimate goal is emmetropia (0 D), a slightly myopic target is traditionally selected to minimize the risk of postoperative hyperopia. The slightly more myopic postoperative outcomes in the RayOne HA group indicate that the intended myopic target (−0.75 to 0.00 D) was closely achieved, whereas PCB00 and Akreos Adapt tended toward plano or mild hyperopia. Therefore, if the aim is true emmetropia, the target refraction for RayOne HA may need to be adjusted toward plano rather than the conventional first-minus. These findings highlight the importance of lens-specific target planning to optimize refractive outcomes.

Although these differences did not reach statistical significance at all time points, the trend was consistent and may be clinically relevant in refractive planning. This suggests that while longitudinal IOL shift was minimal, ELP prediction and lens-specific design features may contribute to refractive differences [10].

The Tecnis PCB00 is a conventional aspheric monofocal IOL that has demonstrated stable distance visual outcomes and predictable postoperative refractive results [18]. In a large retrospective cohort of 1242 eyes implanted with the Tecnis PCB00 IOL, 67.95% achieved a postoperative spherical equivalent within ±0.50 D of target and 93.32% were within ±1.00 D of target refraction [19].

Direct comparative studies on Akreos Adapt and RayOne HA are limited. Previous reports on Akreos Adapt have demonstrated good in-bag centration and minimal rotational instability, supporting stable postoperative performance [20]. Clinical evaluations of RayOne models, including the RayOne EMV enhanced monofocal and RayOne Galaxy lenses, have demonstrated favorable visual outcomes and predictable postoperative performance, supporting the reliability of the RayOne platform [21,22]. These data contextualize our results, in which RayOne HA achieved slightly more myopic outcomes, consistent with the target refractive planning.

Recent analyses suggest that refractive differences may not be fully explained by axial IOL movement alone. While longitudinal shift was similar across groups in the present study, changes in ELP and IOL design characteristics have been identified as factors that influence refractive outcomes even in the absence of measurable physical shift [16]. Such variations may arise from differences in haptic configuration, optic geometry, and capsular bag interaction, which can affect the final lens position relative to the predicted ELP, and thus modify refractive results.

Several mechanisms may contribute to the greater myopic deviation observed with RayOne HA lenses compared with PCB00 and Akreos Adapt, despite similar optic and haptic designs. Both RayOne HA and Akreos Adapt are made of hydrophilic acrylic materials, characterized by a relatively high water content and lower refractive index compared with typical hydrophobic acrylic lenses. However, differences in specific material formulations may influence postoperative behavior. Hydrophilic acrylic IOLs can be more compressible during implantation, and this viscoelastic property may result in slight anterior displacement or compression of the optic within the capsular bag, potentially contributing to a small myopic shift (e.g., ~−0.2 D). Additionally, hydrophilic materials are capable of absorbing additional water during the postoperative healing period, which could lead to minimal increases in effective lens power over time that subtly influence refractive outcomes [23]. Material elasticity and haptic–capsular bag interaction may introduce further variation in postoperative anterior–posterior lens position, even when surgical technique is consistent. Differences in ELP prediction due to IOL constants or the performance of biometry formulas for lenses with distinct material properties may also lead to systematic deviations from target refraction, despite using identical calculation methods [24]. The proprietary Cornerstone design of RayOne HA aims to enhance intraocular stability by balancing haptic forces and controlling injector exit and vaulting. While this primarily targets positional stability, its effect on subtle variations in ELP and refractive predictability has not been extensively studied. Another contributing factor is haptic design. Haptics provide mechanical support for centration and stabilization of the optic. Variations in haptic configuration can influence decentration and tilt, and therefore subtle refractive shifts. Akreos Adapt utilizes a four-point/plate-type haptic design, whereas PCB00 and RayOne HA employ C-loop designs with two main contact points. Studies using anterior segment imaging have demonstrated that plate-haptic IOLs tend to exhibit less decentration and tilt than C-loop IOLs, suggesting that broader capsular contact enhances positional stability [25]. These material, design, and biomechanical characteristics likely explain why the RayOne HA lenses in our cohort achieved postoperative refractions closely aligned with their intended slight myopic target, showing minimal deviation from the planned outcome, whereas PCB00 and Akreos Adapt lenses tended to target emmetropia.

Visual acuity outcomes further support these findings. BCVA was comparable across all groups at all time points, indicating that optical quality and anatomic placement were adequate regardless of IOL type. UCVA tended to be slightly lower in the RayOne HA group at certain follow-ups, which may be explained by the more myopic refractive outcomes in this group rather than by any difference in IOL stability. This highlights the importance of refractive targeting in interpreting UCVA results, as even small deviations from emmetropia can influence uncorrected vision.

Overall, patient satisfaction was high across all groups, reflecting that small differences in postoperative refraction did not significantly impact perceived visual quality.

These insights reinforce the notion that refractive predictability is multifactorial, involving both biometric prediction accuracy and design-related influences.

This study has several limitations. First, although consecutive eligible patients were prospectively enrolled, the study included a relatively limited sample size without a formal a priori sample size calculation and evaluated only three monofocal IOL models. Second, the follow-up period was limited to six months. Third, only longitudinal axial IOL position was assessed, and potential tilt or decentration was not analyzed. The study included only routine cataract cases, and the results may not be directly applicable to eyes with previous ocular surgery or significant ocular comorbidities. Finally, these findings may not be directly generalizable to other IOL types or materials.

## 5. Conclusions

In our cohort, all three monofocal IOLs demonstrated comparable and clinically stable in-the-bag positioning over the first six postoperative months, with only minimal longitudinal shift. Despite this positional stability, small but consistent differences in postoperative refractive outcomes were observed, indicating that IOL design and ELP prediction may influence refractive predictability independent of lens position. From a clinical perspective, our findings suggest that targeting slight myopia when implanting certain IOL designs may improve the likelihood of achieving emmetropia. Specifically, when implanting the RayOne HA IOL, targeting plano rather than the conventional first-minus target may improve postoperative refractive accuracy. Accurate prediction of postoperative refractive outcomes remains one of the key goals of modern cataract surgery, particularly as patient expectations continue to increase. Tailoring target refraction according to the implanted IOL type may further enhance refractive predictability and patient satisfaction, ensuring optimal visual outcomes for patients.

## Figures and Tables

**Table 1:** Key characteristics of implanted intraocular lenses relevant to in-bag stability and effective lens position.

Feature	PCB00 (Johnson & Johnson)	Akreos Adapt (Bausch + Lomb)	RayOne HA (Rayner)
Material	Hydrophobic acrylic	Hydrophilic acrylic	Hydrophilic acrylic
Refractive index	~1.47	~1.46	~1.46
Optic design	Aspheric biconvex, 6.0 mm optic	Aspheric biconvex, 5.7–6.0 mm optic	Aspheric biconvex, 6.0 mm optic
Haptics	Single-piece C-loop	4-plate haptic design	Single-piece C-loop
Haptic angulation	0°	0°	0°
Overall length	13.0 mm	10.5 mm	11.4 mm
A-constant (SRK/T)	~119.3	~118.4	~118.6
Edge design	Sharp posterior square edge	Square edge	Sharp posterior square edge

**Table 2:** Baseline demographic and clinical characteristics.

	PCB00 (N = 36)	Akreos Adapt (N = 31)	RayOne HA (N = 27)	Total (N = 94)	*p* Value
Age (years)					0.122 ^1^
Mean (SD)	75.5 (7.2)	77.2 (6.8)	73.0 (9.2)	75.3 (7.8)	
Range	54.0–93.0	64.0–93.0	55.0–89.0	54.0–93.0	
Gender					0.601 ^2^
Male	14.0 (38.9%)	11.0 (35.5%)	13.0 (48.1%)	38.0 (40.4%)	
Female	22.0 (61.1%)	20.0 (64.5%)	14.0 (51.9%)	56.0 (59.6%)	
IOL power (D)					0.306 ^1^
Mean (SD)	21.9 (2.5)	21.5 (2.8)	21.0 (1.2)	21.5 (2.3)	
Range	16.0–26.0	16.5–26.0	18.0–23.0	16.0–26.0	
Target refraction (D)					0.438 ^1^
Mean (SD)	−0.3 (0.2)	−0.3 (0.1)	−0.2 (0.2)	−0.3 (0.2)	
Range	−0.6–0.0	−0.6–0.0	−0.6–0.0	−0.6–0.0	
Cylinder (D)					0.487 ^1^
Mean (SD)	0.7 (0.4)	0.6 (0.5)	0.8 (0.5)	0.7 (0.5)	
Range	0.0–1.6	0.0–1.9	0.0–2.0	0.0–2.0	
AL (mm)					0.075 ^1^
Mean (SD)	23.6 (0.8)	23.3 (1.1)	23.8 (0.8)	23.6 (0.9)	
Range	22.1–26.2	21.5–25.8	22.5–24.9	21.5–26.2	
ACD (mm)					0.062 ^1^
Mean (SD)	3.1 (0.3)	3.1 (0.4)	3.2 (0.3)	3.1 (0.4)	
Range	2.5–3.6	2.3–3.8	2.7–4.0	2.3–4.0	
LT (mm)					0.764 ^1^
Mean (SD)	4.5 (0.4)	4.5 (0.7)	4.4 (0.4)	4.5 (0.5)	
Range	3.8–5.2	1.3–5.2	3.6–5.1	1.3–5.2	
CCT (µm)					0.403 ^1^
Mean (SD)	544.8 (33.7)	523.7 (101.6)	540.4 (41.8)	536.6 (65.8)	
Range	439.0–613.0	440.0–613.0	456.0–612.0	440.0–613.0	
WTW (mm)					0.111 ^1^
Mean (SD)	12.0 (0.3)	11.9 (0.3)	12.2 (0.4)	12.0 (0.4)	
Range	11.6–12.4	11.5–12.5	11.4–12.9	11.4–12.9	

SD—standard deviation; D—diopter; IOL—intraocular lens; AL—axial length; ACD—anterior chamber depth; LT—lens thickness; CCT—central corneal thickness; µm—micron; WTW—white-to-white corneal diameter; mm—millimeter; ^1^—Linear Model ANOVA; ^2^—Pearson’s Chi-squared test.

**Table 3:** In-the-bag stability of 3 types of IOLs at 1 week, 1 month, 6 months and difference.

	PCB00 (N = 36)	Akreos Adapt (N = 31)	RayOne HA (N = 27)	Total (N = 94)	*p* Value
IOL position (1 w) µm					0.149 ^1^
Mean (SD)	4136.1 (391.1)	3990.0 (259.8)	4116.7 (276.4)	4082.3 (324.0)	
Range	3450.0–5510.0	3360.0–4670.0	3580.0–4680.0	3360.0–5510.0	
IOL position (1 mo) µm					0.730 ^1^
Mean (SD)	4005.2 (352.5)	4015.2 (313.0)	4104.8 (311.8)	4037.1 (350.2)	
Range	3880.0–4990.0	3280.0–4760.0	3400.0–4590.0	3280.0–4990.0	
SHIFT (1 w–1 mo) µm					0.535 ^1^
Mean (SD)	35.3 (212.4)	−24.8 (259.0)	17.0 (184.6)	10.2 (220.9)	
Range	−270.0–520.0	−710.0–590.0	−350.0–450.0	−710.0–590.0	
IOL position (6 mo) µm					0.295 ^1^
Mean (SD)	4045.0 (286.6)	3953.5 (305.4)	4069.3 (315.9)	4021.8 (302.2)	
Range	3550.0–4990.0	3510.0–5000.0	3590.0–4760.0	3510.0–5000.0	
SHIFT (1 mo–6 mo) µm					0.781 ^1^
Mean (SD)	80.0 (241.8)	73.2 (293.0)	35.6 (243.3)	65.0 (258.0)	
Range	−370.0–870.0	−480.0–730.0	−370.0–540.0	−480.0–870.0	

IOL—intraocular lens; µm—micron; SD—standard deviation; ^1^—Linear Model ANOVA.

**Table 4:** Differences in visual function at 1 week, 1 month and 6 months between 3 groups.

	PCB00 (N = 36)	Akreos Adapt (N = 31)	RayOne HA (N = 27)	Total (N = 94)	*p* Value
Spheric equivalent post.op. (PR 1 w)					0.296 ^1^
Mean (SD)	−0.2 (0.6)	−0.2 (0.4)	−0.4 (0.8)	−0.3 (0.6)	
Range	−1.6–0.8	−1.6–0.2	−2.6–0.6	−2.6–0.8	
Spheric equivalent post.op. (PR 1 mo)					0.085 ^1^
Mean (SD)	−0.2 (0.6)	−0.2 (0.4)	−0.5 (0.8)	−0.3 (0.6)	
Range	−1.8–0.8	−1.5–0.4	−2.4–1.2	−2.4–1.2	
Spheric equivalent post.op. (PR 6 mo)					0.156 ^1^
Mean (SD)	−0.2 (0.5)	−0.1 (0.6)	−0.3 (0.7)	−0.2 (0.6)	
Range	−1.6–0.8	−1.5–1.5	−1.8–1.0	−1.8–1.5	
Postoperative UCVA 1 w					0.143 ^1^
Mean (SD)	0.7 (0.3)	0.8 (0.2)	0.7 (0.3)	0.7 (0.2)	
Range	0.1–1.0	0.4–1.0	0.1–1.0	0.1–1.0	
Postoperative BCVA 1 w					0.312 ^1^
Mean (SD)	0.9 (0.2)	0.9 (0.1)	0.9 (0.2)	0.9 (0.2)	
Range	0.4–1.0	0.5–1.0	0.5–1.0	0.4–1.0	
Postoperative UCVA 1 mo					0.084 ^1^
Mean (SD)	0.8 (0.3)	0.8 (0.2)	0.7 (0.3)	0.8 (0.3)	
Range	0.3–1.0	0.3–1.0	0.1–1.0	0.3–1.0	
Postoperative BCVA 1 mo					0.345 ^1^
Mean (SD)	0.9 (0.2)	1.0 (0.0)	0.9 (0.1)	0.9 (0.1)	
Range	0.0–1.0	0.8–1.0	0.6–1.0	0.0–1.0	
Postoperative UCVA 6 mo					0.296 ^1^
Mean (SD)	0.8 (0.2)	0.8 (0.2)	0.7 (0.3)	0.8 (0.2)	
Range	0.1–1.0	0.4–1.0	0.1–1.0	0.1–1.0	
Postoperative BCVA 6 mo					0.890 ^1^
Mean (SD)	1.0 (0.1)	1.0 (0.1)	1.0 (0.1)	1.0 (0.1)	
Range	0.7–1.0	0.6–1.0	0.5–1.0	0.5–1.0	

PR—postoperative refraction; UCVA—uncorrected visual acuity; BCVA—best corrected visual acuity; SD—standard deviation; and ^1^—Linear Model ANOVA.

**Table 5:** Differences in the margin of error of postoperative refraction (SE—target refraction) at week 1, month 1 and month 6 between 3 groups.

	PCB00 (N = 36)	Akreos Adapt (N = 31)	RayOne HA (N = 27)	Total (N = 94)	*p* Value
Margin of error 1 w					0.066 ^1^
Mean (SD)	0.1 (0.5)	0.1 (0.5)	−0.3 (0.9)	−0.0 (0.6)	
Range	−0.8–0.9	−1.6–0.7	−1.6–0.6	−1.6–0.9	
Margin of error 1 mo					0.004 ^1^
Mean (SD)	0.1 (0.5)	0.1 (0.4)	−0.3 (0.8)	−0.0 (0.6)	
Range	−1.1–1.0	−1.4–0.6	−1.8–0.9	−1.8–1.0	
Margin of error 6 mo					0.045 ^1^
Mean (SD)	0.1 (0.4)	0.2 (0.6)	−0.2 (0.7)	0.1 (0.6)	
Range	−0.8–1.0	−1.1–2.0	−1.8–1.3	−1.8–2.0	

W—week; mo—month; SD—standard deviation; ^1^—Linear Model ANOVA.

**Table 6:** Differences in patient satisfaction after one and six months on Likert scale from 0 (worst satisfaction) to 5 (excellent satisfaction).

	PCB00 (N = 36)	Akreos Adapt (N = 31)	RayOne HA (N = 27)	Total (N = 94)	*p* Value
Patient satisfaction 1 mo					0.059 ^1^
Mean (SD)	4.5 (1.2)	4.8 (0.4)	4.2 (0.8)	4.5 (0.9)	
Range	0.0–5.0	4.0–5.0	2.0–5.0	0.0–5.0	
Patient satisfaction 6 mo					0.978 ^1^
Mean (SD)	4.6 (0.7)	4.6 (0.8)	4.6 (0.5)	4.6 (0.7)	
Range	2.0–5.0	2.0–5.0	4.0–5.0	2.0–5.0	

Mo—month; SD—standard deviation. ^1^—Linear Model ANOVA.

## Data Availability

The data presented in this study are available upon request from the corresponding author. The data are not publicly available due to privacy and ethical restrictions.

## Abbreviations

The following abbreviations are used in this manuscript:

IOL	Intraocular lens
ELP	Effective lens position
UCVA	Uncorrected distance visual acuity
BCVA	Best-corrected distance visual acuity
SE	Spherical equivalent
ACD	Anterior chamber depth
LT	Lens thickness
WTW	White-to-white
AL	Axial length

## Appendix A

**Table A1:** Percentage of eyes within target refraction at final follow-up.

Outcome	PCB00 (N = 36)	Akreos Adapt (N = 31)	RayOne HA (N = 27)
Eyes within ±0.50 D	25 (69.4%)	22 (71.0%)	17 (63.0%)
Eyes within >±0.50 to ±1.00 D	9 (25.0%)	6 (19.4%)	7 (25.9%)
Eyes within >±1.00 D	2 (5.6%)	3 (9.7%)	3 (11.1%)

D—diopter.
